# Apical-basal polarity regulators are essential for slit diaphragm assembly and endocytosis in *Drosophila* nephrocytes

**DOI:** 10.1007/s00018-021-03769-y

**Published:** 2021-03-02

**Authors:** Stefanie Heiden, Rebecca Siwek, Marie-Luise Lotz, Sarah Borkowsky, Rita Schröter, Pavel Nedvetsky, Astrid Rohlmann, Markus Missler, Michael P. Krahn

**Affiliations:** 1 Medical Cell Biology, Medical Clinic D, University Hospital of Münster, Albert-Schweitzer Campus 1-A14, 48149 Münster, Germany; 2 Institute of Anatomy and Molecular Neurobiology, University of Münster, Vesaliusweg 2-4, 48149 Münster, Germany

**Keywords:** Nephrocyte, Podocyte, Slit diaphragm, Polarity, PAR-3, aPKC, PAR-1, Expansion microscopy

## Abstract

Apical-basal polarity is a key feature of most epithelial cells and it is regulated by highly conserved protein complexes. In mammalian podocytes, which emerge from columnar epithelial cells, this polarity is preserved and the tight junctions are converted to the slit diaphragms, establishing the filtration barrier. In *Drosophila*, nephrocytes show several structural and functional similarities with mammalian podocytes and proximal tubular cells. However, in contrast to podocytes, little is known about the role of apical-basal polarity regulators in these cells. In this study, we used expansion microscopy and found the apical polarity determinants of the PAR/aPKC and Crb-complexes to be predominantly targeted to the cell cortex in proximity to the nephrocyte diaphragm, whereas basolateral regulators also accumulate intracellularly. Knockdown of PAR-complex proteins results in severe endocytosis and nephrocyte diaphragm defects, which is due to impaired aPKC recruitment to the plasma membrane. Similar, downregulation of most basolateral polarity regulators disrupts Nephrin localization but had surprisingly divergent effects on endocytosis. Our findings suggest that morphology and slit diaphragm assembly/maintenance of nephrocytes is regulated by classical apical-basal polarity regulators, which have distinct functions in endocytosis.

**Supplementary Information:**

The online version contains supplementary material available at 10.1007/s00018-021-03769-y.

## Introduction

Mammalian podocytes are essential for the establishment and function of the filtration barrier and impaired function or loss of these cells causes many renal diseases such as diabetic nephropathy, nephrotic syndrome and focal segmental glomerulosclerosis (FSGS). Although much knowledge has been derived from mouse and zebrafish, podocyte research is hampered by the lack of suitable cell culture models, in particular for the investigation of the underlying pathomechanisms and the development of therapeutic approaches.

To address this problem, *Drosophila* nephrocytes have been emerged as a model system for the mammalian kidney, comprising the filtration component (analog to podocytes) and the endocytosis and modification unit (analog to proximal tubules). Nephrocytes develop from myoblasts and are found in two populations, which float in the hemolymph: Garland nephrocytes surrounding the proventriculus and pericardial nephrocytes lining up along the heart tube. Both cell types show similar ultrastructural and functional characteristics [1, 2]. The plasma membrane forms deep invaginations, which we have shown recently to be mostly not dead-ended lacunae, but channels, spanning from the surface of the nephrocyte into the body and back to another spot on the surface [3]. Homologues of mammalian Cubilin and Amnionless, which are important receptors for endocytosis, are localized at these lacunae/channels, suggesting this part to be analog to the (proximal) tubule system in mammals [4]. Similar to their mammalian counterparts, nephrocytes establish a filtration barrier by forming a slit diaphragms, which demarcate the channels from the extracellular space in a size-selective manner. These slit diaphragms composed of homologues of mammalian Nephrin (Sticks and stones, Sns and Hibris, Hbs) and Neph1 (Kin of irre, Kirre and Roughest), which are stabilized by the Podocin homologue Mec2. Furthermore, the transmembrane protein Crumbs (Crb) and its intracellular adaptor protein Stardust (Sdt, Pals1 in mammals) accumulate at nephrocyte diaphragms and are essential for the establishment of the nephrocyte diaphragm and regulate endocytosis by modulating the localization and activation of the FERM-domain protein Moesin [5, 6]. Notably, in humans, mutations in Crb2 result in filtration defects and nephrotic syndrome [7, 8] and wild-type Crb2 but none of these patient mutations can compensate for downregulation of *Drosophila* Crb in nephrocytes [5].

Due to their high degree of structural and functional similarities to mammalian podocytes, the genetic tools available in *Drosophila* and the short generation time and easy handling, nephrocytes have been used as a model to mimic podocyte diseases in several studies (reviewed by [9–11]). Nephrocytes were used to imitate the phenotypes of steroid-resistant nephrotic syndrome caused by mutations in the Rab11 regulator TBC1D8B [12], the Rab5-interacting proteins GAPVD1 and ANKFY1 [13] and the RhoA regulator KANK [14]. Furthermore, the authors used nephrocytes to model nephrotic syndrome caused by mutations in distinct genes by downregulating the expression of these genes using RNAi [15]. With this approach, they were able to elucidate the pathomechanism of mutations in the gene encoding for co-enzyme Q10, which are associated with enhanced production of reactive oxygen species, leading to loss of nephrocyte diaphragms. A similar approach was used by Fu et al., screening 40 genes associated with nephrotic syndrome in nephrocytes [16]. We have recently analyzed the impact of patient mutations associated with FSGS in the Actin-modulating protein INF2 on Actin dynamics and Nephrin localization in nephrocytes (Bayraktar et al., *JASN* in press). Variants of the Apolipoprotein 1 (APOL-1) are associated with kidney failure due to podocyte damage but the underlying pathomechanism is still unclear. Expression of APOL-1 variants in nephrocytes revealed a possible link between these risk variants and acidification of organelles [17, 18]. Although *Drosophila* has no closed vascular system but an open heart, which circulates the hemolymph throughout the body cavity of the larvae and fly, Hartley et al. identified a function of pericardial nephrocytes in protecting from cardiomyopathy, presumably by modulating the levels of circulating heart-protective factors [19]. Furthermore, clearance of peptidoglycan from the hemolymph by nephrocytes is essential for immune functions of the fly [20].

Finally, nephrocytes have been shown to mimic diabetic nephropathy with loss of Nephrin expression if larvae are fed with a high sucrose diet [21], which might facilitate the screening for therapeutic approaches for the treatment of this disease.

Throughout the development of the mammalian kidney, podocytes differentiate from columnar epithelial cells, maintaining their apical-basal polarity. During this process, the tight junctions (TJ) are converted to the slit diaphragms and the apical domain is dramatically enlarged at the expense of the basolateral membrane, which is reduced to the contact zone of the foot processes with the glomerular basement membrane. For podocytes it has already been shown, that protein complexes, which are essential for TJ assembly and -function, are also important regulators of slit diaphragm assembly/maintenance. One example is the adaptor protein *Zonula Occludens* protein 1 [22, 23], whose *Drosophila* homologue Polychaetoid (Pyd) has recently been confirmed to regulate slit diaphragm assembly in nephrocytes, too [24]. PARtitioning defective 3 (PAR-3) and atypical Protein Kinase C (aPKC) both control TJ formation and are also involved in slit diaphragm assembly [25–30].

In classical epithelia, the PAR/aPKC complex (PAR-3, aPKC and PAR-6) and the Crb-complex (Crb, Pals1/Sdt, PATJ) determine the identity of the apical plasma membrane, which is counterbalanced by the lateral scaffolding proteins Discs Large (Dlg), Lethal (2) Giant Larvae (Lgl) and Scribble (Scrb) as well as by the kinases PAR-1 and LKB1 (for review see [31–33]). Reciprocal exclusion from the respective “wrong” domains via phosphorylation of aPKC/PAR-3 by PAR-1 and conversely of PAR-1/Lgl by aPKC is essential for apical-basal polarity and disturbed protein expression or phosphorylation results in its loss.

Despite their use as a podocyte model in biomedical research, little is known about basic cell biological characteristics of *Drosophila* nephrocytes. We have recently described a role for the Crb-complex in nephrocyte diaphragm establishment and endocytosis in garland nephrocytes [5, 6]. Otherwise nothing is known about the function of apical-basal polarity regulators in this cell type and whether nephrocytes exhibit an apical-basal polarity similar to mammalian podocytes. Therefore, the aim of this study was to characterize the precise subcellular localization of polarity regulators and their function in *Drosophila* nephrocytes. Using expansion microscopy, we were able to visualize individual nephrocyte diaphragms by labeling the Nephrin homologue Sns. Co-stainings reveal that the apical determinants Bazooka (the *Drosophila* homologue of PAR-3 in mammals), aPKC, PAR-6, Crb, Stardust and PATJ are all targeted to the cell cortex, partly overlapping with the slit diaphragm marker. In contrast, basolateral polarity determinants (Dlg, Lgl, PAR-1, and LKB1) are only partly localized to junctional complexes but also accumulate in vesicular structures inside of nephrocytes.

RNAi-mediated knockdown of PAR-complex proteins results in severe slit diaphragm and endocytosis defects and rescue experiments identified membrane targeting of aPKC as important for these processes. Regarding lateral polarity regulators, downregulation of each tested candidate disturbs cortical Nephrin localization but only knockdown of Scribble also leads to severe reduction in endocytosis.

## Materials and methods

### *Drosophila* stocks and genetics

Fly stocks were cultured on standard cornmeal agar food and maintained at 25 °C. For evaluation of filtration efficiency, a fly line expressing 2xGFP fused to the secretion signal of Atrial natriuretic peptide under a ubiquitous promoter (*ubi*::ANP-2xGFP) was established, combined with the nephrocyte-specific driver line *sns::GAL4* [2] and subsequently crossed to the responder lines. For downregulation of specific genes for immunostainings and electron microscopy, *sns::GAL4* without *ubi*::ANP-GFP-GFP was used. For all RNAi experiments, crosses were kept for 3 days at 25 °C and larvae subsequently shifted to 29 °C to obtain maximum expression. The following RNAi-lines were used in this study: UAS::aPKC-RNAi (#34332), UAS::Dlg-RNAi (#34854), UAS::LKB1-RNAi (#34362), UAS::mCherry-RNAi (#35785), UAS::PAR-1-RNAi (#32410), UAS::Scrb-RNAi (#39073) (all obtained from Bloomington Stock Center, Bloomington, IL, USA), UAS::Kirre-RNAi (#109585), UAS::Lgl-RNAi (#109604), UAS::Or83b-RNAi (negative control, #100825), UAS::PAR-6 (#19730) (provided by Vienna *Drosophila* Resource Center, Austria). The GFP-PAR-1 trap was provided by D. St. Johnston (Carnegie CC01981, [34]) and GFP-LKB1 is a transgene containing a genomic fragment of *lkb1* and a GFP inserted before the start codon [35]. UASt::GFP-Baz and UASt::GFP-Baz S980A were described in Krahn et al. 2010. The UAS::Baz-shRNA (shRNA: 5′-GCGAAACAGAAACCAAGAGAG-3′ cloned in pWalium) line was established in this study using the Phi-C31-Integrase system with attP-VK00002 (28E). For Baz rescue experiments, *sns::GAL4* was recombined with *UAS::Baz-shRNA.* UAS::aPKC-PH(PLCδ) was cloned by fusing the PH domain of PLCδ to the C-terminus of aPKC and the transgene was established in attP86F. UAS::aPKC-CAAX was provided by Sol Sotillos [36] and UAS::aPKC∆N was obtained from Bloomington stock center (#51673).

### Endocytosis assays

For the ANP-2xGFP accumulation assay, garland nephrocytes from wandering third instar larvae were microdissected in HL3.1 saline [37], fixed in 4% PFA in PBS for 10 min, stained with DAPI for 20 min, washed with PBS, and mounted in Mowiol. ANP-2xGFP accumulation per nephrocyte area (CTCF = Corrected Total Cell Fluorescence) was analyzed and quantified with ImageJ after subtracting the autofluorescent background of dissected larvae. For each genotype, at least 75 nephrocytes of 10 independent larvae were quantified.

For FITC-Albumin endocytosis assays, garland nephrocytes were dissected as described above and incubated with 1 mg/ml FITC-Albumin (SIGMA #A9771) in HL3.1 saline for 1 min. After two washes with PBS, cells were fixed and processed as described above. For each genotype, at least 15 independent larvae (> 50 nephrocytes total) were quantified.

### Immunohistochemistry

Garland nephrocytes were dissected as described above and heat-fixed for 20 s in boiling heat fix saline (0.03% Triton X-100). Subsequently, nephrocytes were washed three times in PBS + 0.1% Triton X-100 and blocked with 1% BSA for 1 h, incubated over night with primary antibodies in PBS + 0.1% Triton X-100 + 1% BSA, washed three times and incubated for 2 h with secondary antibodies. After three washing steps and DAPI-staining, nephrocytes were mounted with Mowiol. Primary antibodies used were rabbit anti-aPKC (1:500, #sc-216, Santa Cruz), rabbit anti-Baz (1:500, [38]), mouse anti-Crb (1:10, Cq4, Development Study Hybridoma Bank, DSHB), mouse anti-Dlg (1:10, 4F3, DSHB), goat anti-GFP (1:500, #600-101-215, Rockland), mouse anti-GM130 (1:200, #610822, BD Biosciences), mouse anti-GS28 (1:200, #611185, BD Bioscience), mouse anti-Hrs (1:10, 27–4, DSHB), mouse anti-Integrin β1 (1:20, CF.6G11, DSHB), guinea pig anti-Lgl (1:500, [39]), guinea pig anti-Megalin (1:100, [40]), rat anti-PAR-6 (1:500, [39]), rabbit anti-PAR-6 (1:500, this study), guinea pig anti-PATJ (1:500, [41]), mouse anti-p230 (1:200, #611280, BD Bioscience), rabbit anti-Rab5 (1:500, #2143, Cell signaling), mouse anti-Rab7 (1:20, DSHB), mouse anti-Rab11 (1:500, #61065, BD Biosciences), guinea pig anti-Rab5 (1:1000), rabbit anti-Rab7 (1:1000) and rabbit anti-Rab11 (1:1000) were kindly provided by A. Nakamura [42], mouse anti-Sdt (1:20, [43]), chicken anti-Sns [1:1000, 5], mouse anti-Talin (1:20, E16B, DSHB), mouse anti-Vti1b (1:500, #611404, BD Bioscience). An antibody against *Drosophila* Vinculin was raised by immunizing rabbits with purified full-length GST-Vinculin isolated from *E. coli* (Eurogentec) and used 1:500. Secondary antibodies conjugated with Alexa 488, Alexa 568 and Alexa 647 (Life technologies) were used at 1:400. Images were taken on a Leica SP8 confocal microscope using lightning program and processed using ImageJ. Colocalization was quantified using the Pearson correlation coefficient.

### Expansion microscopy

Expansion microscopy was adapted from [44, 45]. Garland nephrocytes were dissected and stained like described above but with a twofold higher concentration primary antibodies and a fourfold higher concentration of secondary antibodies. Instead of Alexa 647, we used ATTO 647. After staining, the samples were labeled with 1 M MA-NHS in DMSO (diluted in PBS 1:1000) for 2 h and subsequently incubated with monomer solution (1 × PBS, 2 M NaCl, 8.625% (w/v) sodium acrylate, 2.5% (w/v) acrylamide, 0.15% (w/v) *N*, *N*′-methylenebisacrylamide) for 30 min on ice in the dark. After activating monomer solution with 0.2% TEMED, 0.01% 4-hydroxy-TEMPO and 0.2% APS, the samples are transferred to the activated solution in an Ibidi^®^ imaging chamber and covered with a coverslip packed in parafilm. The gelation takes place at 37 °C for 2 h in the dark before the coverslip is removed. Next, the gel was incubated with the digestion buffer (50 mM Tris (pH 8), 1 mM EDTA, 0.5% Triton X-100, 0.8 M guanidine HCl) freshly supplemented with Proteinase K (16 units/mL) over night at 37 °C. Finally, the gel was expanded with water three times for 20 min and subsequently imaged.

### Transmission electron microscopy

Garland nephrocytes of third instar larvae were microdissected in HL3.1 saline, high pressure frozen (EM-PACT2, Leica, Wetzlar, Germany), freeze-substituted in acetone/1% OsO4/5% H_2_O/0.25% uranyl acetate (AFS2, Leica, Wetzlar, Germany) and embedded in Epon. For transmission electron microscopy, 70-nm-thick sections were cut using an ultramicrotome (Leica UC7, Wetzlar, Germany). All samples were imaged with a transmission electron microscope (ZEISS, Libra 120, Germany).

## Results

### Apical polarity regulators are targeted to the cell cortex of nephrocytes

**Fig. 1:**
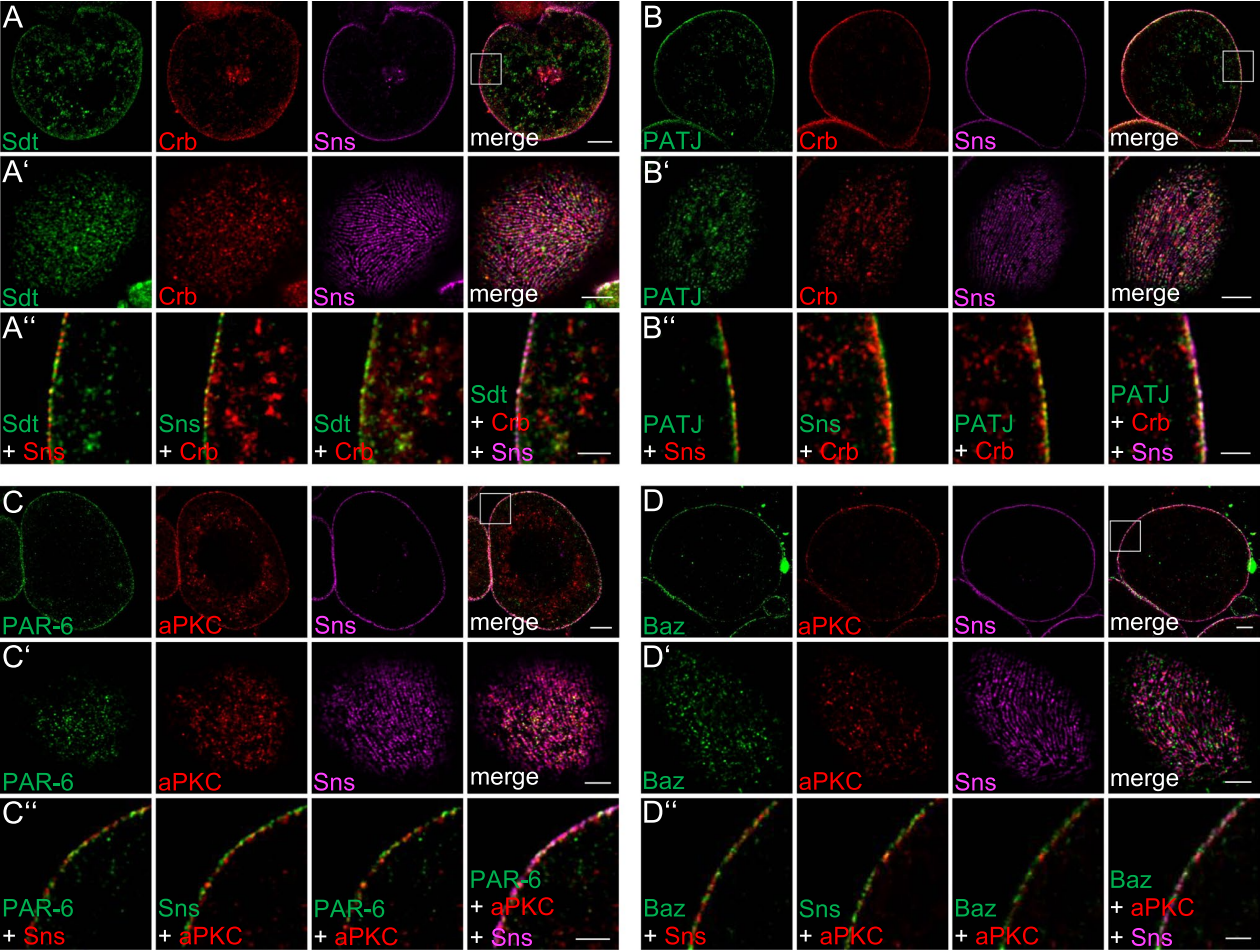
Apical polarity regulators localize at the cortex of nephrocytes. **A**–**D** Garland nephrocytes were dissected from 3rd instar larvae, fixed and stained with the indicated antibodies. Subsequently, samples were embedded in acrylamide gel and subjected to expansion to increase sample size and thus resolution. Two sections were imaged: the equatorial plane (**A**–**D**) and an onview on the surface of the nephrocyte (**A**′–**D**′). **A**″–**D**″ are magnifications of the cortical region from **A**–**D**. Scales bars are 10 µm in **A**–**D**, 5 µm in **A**′–**D**′ and 3 µm in **A**″–**D**″

In epithelial cells, components of the PAR/aPKC and Crb-complex are targeted to the TJ or the apical junctional region in *Drosophila*. In podocytes, at least PAR-3 and aPKC isoforms have been shown to colocalize with slit diaphragm markers [25, 26]. For Crb, Sdt and PATJ, we have already shown a colocalization with Sns at the cortical region of nephrocytes [5, 6]. However, due to the resolution limit and the distance between slit diaphragms in nephrocytes (ca. 300 nm between two slit diaphragms, Supplementary Fig. 3A), the exact localization with respect to slit diaphragms is difficult to evaluate using conventional confocal microscopy. To improve the resolution, we applied expansion microscopy [44, 45] in combination with deconvolution/lightning. By this, we expanded the samples by around 3-fold (2.8-fold), resulting in a dramatically improved resolution which enables us to visualize single slit diaphragms by Sns staining (immunostainings in Fig. 1 compared to electron microscopy in Supplementary Fig. 3A).

Co-staining of Sns with apical polarity regulators revealed a substantial colocalization of Baz, PAR-6 and aPKC as well as of Crb, Sdt and PATJ with Sns at the cell cortex (Fig. 1A–D), although all six proteins do not show the regular finger-print-pattern at the surface as Sns (Fig. 1A’–D’), which represents the slit diaphragm strands. Furthermore, neither Baz/aPKC/PAR-6 nor Crb/Sdt/PATJ exactly colocalize with Sns (Fig. 1A’’–D’’), indicating that the apical junctional complexes are targeted to the cell cortex accumulating to some extent (but not exclusively) at slit diaphragms. This is further underlined by the quantification of Sns-colocalization (Pearson correlation coefficient of 0.25 (Crb), 0.44 (Sdt), 0.54 (PATJ), 0.42 (Baz), 0.49 (PAR-6) and 0.3 (aPKC), respectively). Thus, there is no one-to-one colocalization with Sns at the cell cortex, which would qualify these proteins as essential physical stabilizers of Nephrin/Neph1 at slit diaphragms (as suggested for the mammalian homologues of the PAR/aPKC complex, [25, 26]). Notably, in particular Sdt and aPKC and to a lesser extent Crb, PATJ, PAR-6 and Baz, display an additional vesicular localization inside the nephrocytes, which we do not observe in classical epithelia [46].

### Integrin complexes substantiate the basal plasma membrane domain

**Fig. 2:**
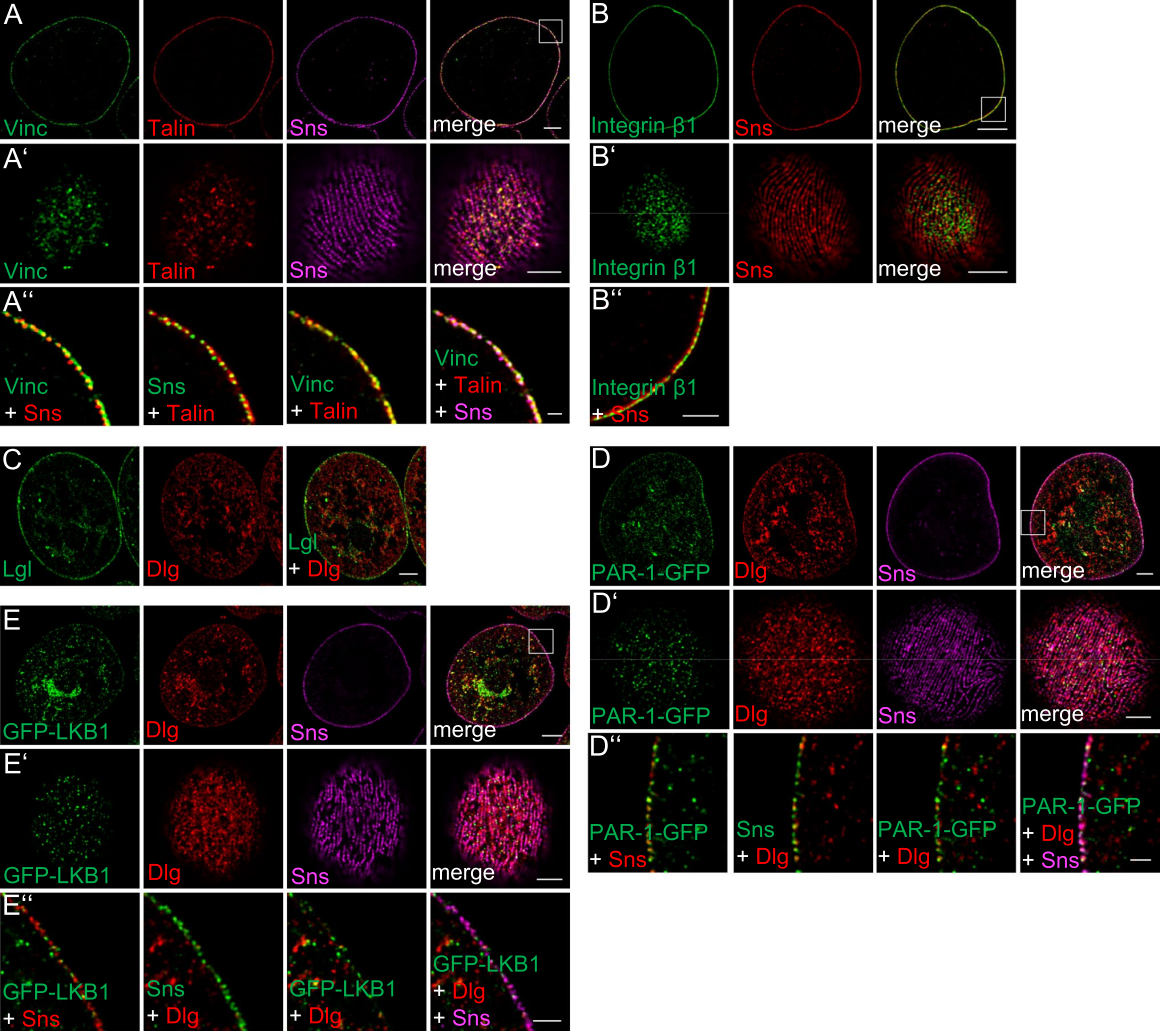
Garland nephrocytes exhibit a basal and a junctional plasma membrane domain. **A**–**E** Garland nephrocytes from 3rd instar larvae in expansion microscopy display a distinct localization of basal Integrin complex members (Integrin β1, Talin and Vinculin) at the plasma membrane between Sns-marked slid diaphragms (**A** and **B**), whereas basolateral cell polarity determinants to some extent accumulate at slit diaphragms but are also found in the vesicular structures in the interior of the nephrocyte (**C**–**E**). Scales bars are 10 µm in **A**–**E**, 5 µm in **A**′–**E**′ and 3 µm in **A**″–**E**″

Podocytes are tethered to the glomerular basement membrane by anchorage of their foot processes via focal adhesions. Expansion microscopy of immunostainings of Vinculin, Talin and Integrin β1 (Myospheroid, Mys in *Drosophila*) in nephrocytes reveal a similar situation with Integrin β1 mostly filling the gap between Sns punctae with some overlap with Sns (Fig. 2A, B, Pearson correlation coefficient is 0.51 [Sns-Vinc), 0.47 (Sns-Talin) and 0.64 (Sns-Integrin)]. This is consistent with the hypothesis that the plasma membrane between slit diaphragms represents the basal membrane domain, in which the Integrin complex anchors the cell to the basement membrane, which enwraps the entire nephrocyte (Supplementary Fig. 3A). However, onviews (Fig. 2A’, B’) demonstrate that none of the basal proteins stains in stripes between the Sns-strands, which we would expect, if Integrin complexes are localized between two slit diaphragms at the entire cortex. Instead, it seems that these proteins form adhesion spots, like focal adhesions in other cell types.

### Nephrocytes do not exhibit a distinct lateral plasma membrane domain

In *Drosophila* (as well as mammalian) epithelial cells, the basolateral cell polarity determinants of the Dlg/Lgl/Scrb complex and the serine/threonine kinases LKB1 and PAR-1 are localized to the lateral plasma membrane and counterbalance the activity of the apical polarity regulators.

Furthermore, in epithelial cells and neural stem cells, at least Dlg and LKB1 show a substantial overlap with the apical junctional PAR/aPKC complex [35, 46], whereas PAR-1/Baz-aPKC as well as Lgl/Baz-aPKC exclude each other and do not colocalize [47–49].

To test, whether nephrocytes exhibit a distinct lateral plasma membrane domain, we used antibody stainings (Dlg. Lgl), a GFP-trap line (PAR-1) and GFP-tagged LKB1 expressed from its endogenous promoter [35] in expansion microscopy.

In contrast to epithelial cells, the basolateral polarity regulators tested are not exclusively targeted to the plasma membrane but also accumulate in vesicular structures in the cell body (Fig. 2C–E). At the cortex, LKB1, Dlg and PAR-1-GFP only display a weak overlapping staining with Sns (Pearson correlation coefficient being 0.29 (LKB1), 0.39 (PAR-1) and 0.3 (Dlg)). Again, we found no one-to-one correlation (Fig. 2D″, E″) and onviews demonstrate that these proteins are not associated with each Sns-marked slit diaphragm strand (Fig. 2D′, E′).

Thus, nephrocytes seem to develop a similar polarity as podocytes with an enlarged apical domain (lacunae and junctional region at slit diaphragms), a lack of a distinct lateral domain and a reduced basal surface (between slit diaphragms, facing the basal membrane). Lateral polarity determinants are partly associated with slit diaphragm complexes but mostly localize in vesicular structures within the cell. Co-stainings of basolateral polarity determinants with markers for early endosomes (Rab5, Hrs), late endosomes/lysosomes (Rab7), recycling endosomes (Rab11), cis-Golgi (GS28, gm130) and trans Golgi network (Vti1b, p230) revealed a colocalization with Vti1b and p230 only for Lgl and a co-staining of GFP-LKB1 with Rab5, indicating an accumulation of Lgl at trans-golgi network tubules and of GFP-LKB1 at early endosomes (Supplementary Figs. 1, 2). For Dlg and PAR-1, we found only a weak colocalization with Rab5 and no substantial colocalization with other markers of vesicular compartments (Supplementary Figs. 1, 2). Notably, we observed some spots with a colocalization of PAR-1-GFP and GFP-LKB1 but not of Dlg with Megalin (Mgl), a component of the endocytosis receptor complex (Supplementary Fig. 2).

### Basolateral polarity regulators are crucial for Sns localization

**Fig. 3:**
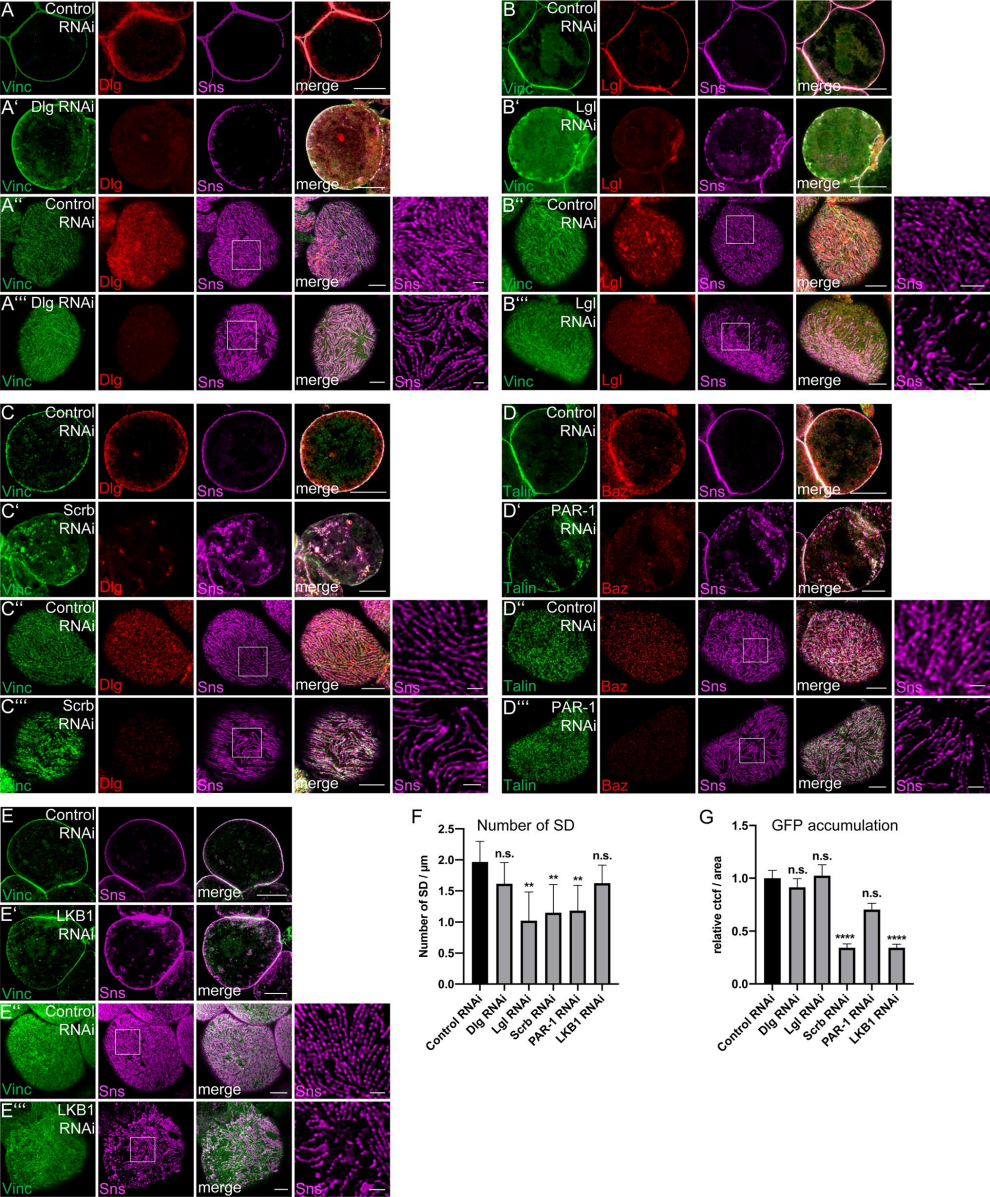
Knockdown of basolateral cell polarity regulators disrupts Sns localization. (**A**–**E**) Garland nephrocytes expressing control shRNA or shRNA/dsRNA against specific basolateral polarity regulators were imaged using confocal microscopy and deconvolution. (**F**) Slit diaphragms (SD)/µm at the surface were quantified. For this, a 5-µm line perpendicular to the Sns-strands was drawn and the number of strands quantified. 5 Lines/nephrocyte and at least 5 nephrocytes were quantified per genotype. **G** Accumulation of secreted ANP-2xGFP in garland nephrocytes of the indicated genotypes was quantified (*n* = 75). Examples of ANP-2xGFP accumulation in nephrocytes expressing indicated shRNA/dsRNAs were depicted in Figure S5. Scale bars are 10 µm in **A**–**E**/**A**′–**E**′ and 5 µm in **A**″–**E**″/**A**′″–**E**′″. Error bars are standard error of the means. Significance was determined by Kruskal–Wallis test and Dunn’s correction: ****p < 0.0001, ***p < 0.001, **p < 0.01,*p < 0.05. n.s. not significant

Although they do not show a one-to-one association with Sns-stained slit diaphragms, knockdown of basolateral polarity determinants results in a strong mislocalization of the slit diaphragm marker Sns (Fig. 3 and Supplementary Fig. 4): In Dlg-, Lgl- and LKB1-knockdown nephrocytes, Sns is partly lost from the cortex and mislocalized to subcortical vesicular structures (Fig. 3A, B, E). An even more dramatic phenotype can be observed in nephrocytes with impaired PAR-1 or Scrb expression: in both cases, Sns is strongly reduced at the cortical rim and redistributed to vesicles all over the cell (Fig. 3C, D). Interestingly, Sns localization at the contact zone between two nephrocytes is less affected, most likely because a higher amount of Sns accumulates in these zones (cp. control stainings). Onviews of these nephrocytes demonstrate gaps in the Sns staining pattern and a decrease of slit diaphragm strands at the surface (Fig. 3A’’’–E’’’), indicating an overall reduction of slit diaphragms (Fig. 3F). Stainings with the basal plasma membrane markers Vinculin or Talin show a disturbed pattern of these proteins similar to that of Sns (Fig. 3A–E), suggesting that impaired Sns localization also disrupts the identity of the basal compartment. Overviews of nephrocyte garlands with knockdown of Dlg/Lgl/Scrb/PAR-1/LKB1 are given in Supplementary Fig. 4, demonstrating a misformation of several nephrocytes as well as fusion phenotypes. Of note, PAR-1 seems to be essential for correct Baz localization as downregulation of PAR-1 results in severe disruption of cortical Baz (Figure S4D). However, this might be an indirect effect of disturbed cortical Sns localization, which is affected in PAR-1-knockdown cells, too.

To test the functional consequences of disturbed Sns localization, we performed two kinds of endocytosis assays: first, ubiquitously expressed and secreted ANP-2xGFP (approximately 54 kDa) is endocytosed by nephrocytes in vivo (examples are given in Supplementary Fig. 5). This assay reflects a steady-state situation and depicts the filtration and endocytosis capacity of nephrocytes (adapted from [50]). Second, incubation of dissected garland nephrocytes with fluorescently labeled Albumin (FITC-Albumin) gives insights into the dynamic filtration and uptake [2, 51–53].

Surprisingly, although Sns mislocalization and its disturbed fingerprint pattern indicates a strong reduction of slit diaphragms, downregulation of Dlg and Lgl does not result in a decreased accumulation of secreted ANP-2xGFP (Fig. 3G and Supplementary Fig. 5A–I). In this endocytosis assay, knockdown of PAR-1 leads to a 25% reduction. Only in case of Scribble- and LKB1-RNAi, a strong reduction (around 70%) in ANP-2xGFP accumulation can be observed (Fig. 3G). Moreover, in short-time endocytosis assays with fluorescently labeled Albumin, no significant differences were detected (Supplementary Fig. 5J). Co-stainings of shRNA/dsRNA-expressing nephrocytes accumulating secreted ANP-2xGFP reveal substantial differences in Rab5 pattern only for Dlg, whereas Rab7-positive vesicles are reduced but its pattern not disturbed in nephrocytes expressing shRNA/dsRNAs against basolateral polarity regulators (Supplementary Fig. 5A–F). Taken together our data demonstrate a critical role for basolateral polarity regulators in establishing/maintaining slit diaphragms in nephrocytes and to some extent in endocytosis, too.

### Apical polarity determinants are essential for slit diaphragm assembly and endocytosis

**Fig. 4:**
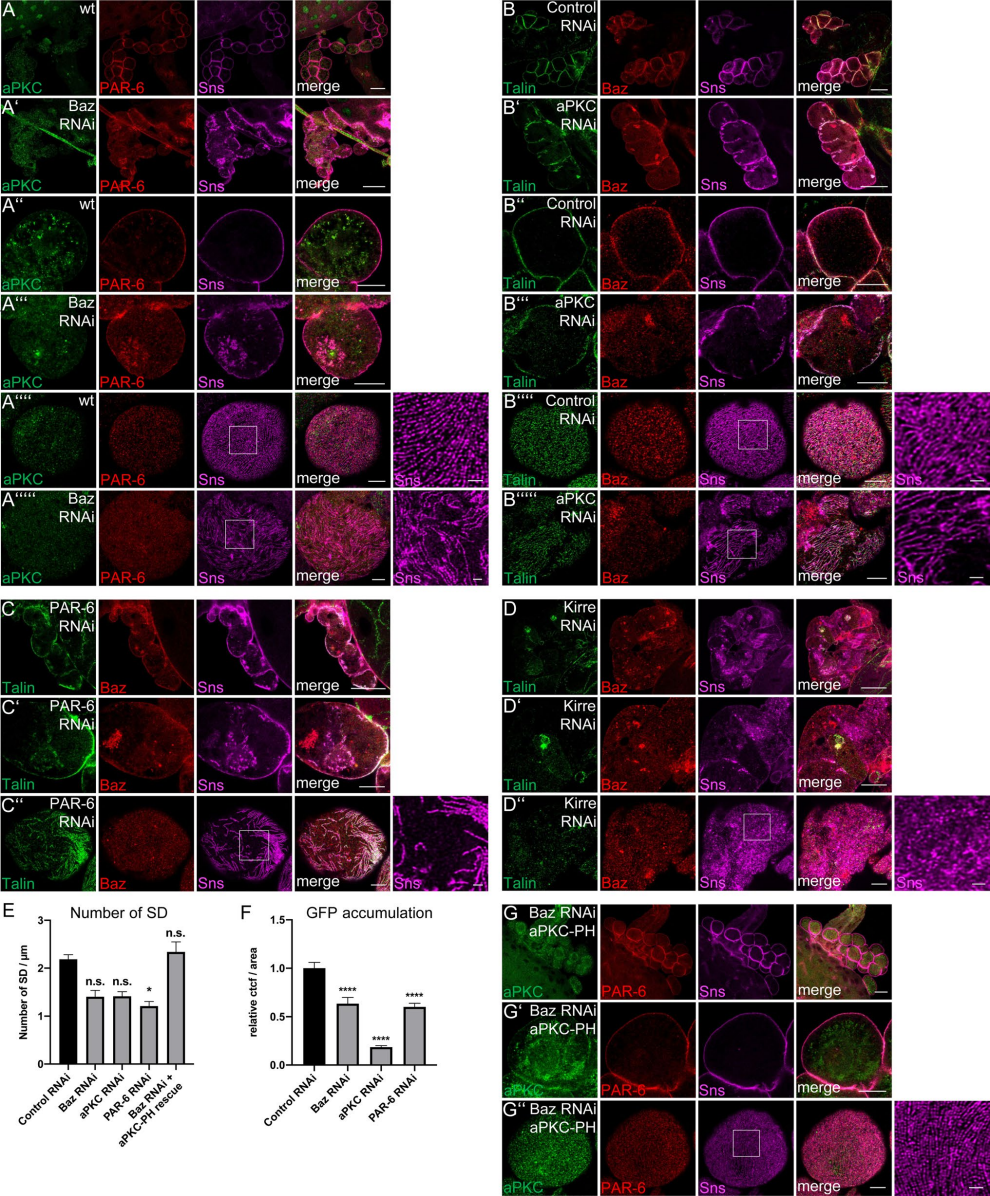
Baz is essential to target aPKC to the cortex of nephrocytes to support slit diaphragm assembly. **A**–**C** Garland nephrocytes expressing shRNA/dsRNA against Baz (**A**), aPKC (**B**) and PAR-6 (**C**). **D** Baz and Sns are mislocalized in nephrocytes expressing Kirre-dsRNA. Similar to knockdown of Baz, nephrocytes are frequently fused upon downregulation of Kirre. **E** Quantification of Sns-marked slit diaphragms in Baz-, aPKC- and PAR-6-shRNA/dsRNA-expressing nephrocytes. Slit diaphragms (SD)/µm at the surface were quantified. For this, a 5-µm line perpendicular to the Sns-strands was drawn and the number of strands quantified. 5 lines/nephrocyte and at least 5 nephrocytes were quantified per genotype. **F** Quantification of ANP-2xGFP accumulation in garland nephrocytes. Examples of ANP-2xGFP accumulation in nephrocytes expressing indicated shRNA/dsRNAs were depicted in Figure S5. **G** Co-expression of aPKC-PH with Baz-shRNA restored cortical PAR-6 and Sns localization as well as the fusion phenotype. Scales bars are 25 µm in **A**/**A**′, **B**/**B**′, **C**, **D** and **G**, 10 µm in **A**″/**A**′″, **B**″/**B**′″, **C**′, **D**′ and **G**′ and 5 µm in **A**″″/**A**′″″, **B**″″/**B**′″″, **C**″, **D**″ and **G**″. Error bars are standard error of the means. Significance was determined by Kruskal–Wallis test and Dunn’s correction: ****p < 0.0001, ***p < 0.001, **p < 0.01,*p < 0.05. n.s. not significant

We have shown recently, that Crb and its adaptor protein Sdt are essential for slit diaphragm assembly and endocytosis in nephrocytes [5, 6]. Crb is found at slit diaphragms but also delineates lacunae and is found on budding vesicles. Mechanistically, Crb’s extracellular domain is essential for slit diaphragm assembly/maintenance, whereas its intracellular FERM-binding motif regulates Moesin localization and activation, thereby controlling endocytosis. For the PAR/aPKC complex, a function in Nephrin/Neph1 stabilization at slit diaphragms has been proposed for podocytes [26, 48], but was never tested in vivo due to technical limitations. We, therefore, used RNAi to downregulate the expression of Baz, aPKC and PAR-6 in garland nephrocytes. As expected, impaired expression of these proteins results in disturbed Sns localization at the cortex of nephrocytes (Fig. 4A–C) and quantification of Sns-positive strands at the surface of nephrocytes reveals a reduction in cells with downregulation of either Baz-, aPKC- or PAR-6 (Fig. 4E). Further analyses using high-pressure freezing and subsequent electron microscopy [3] demonstrate a significant reduction of slit diaphragms and ectopic slit diaphragms located deeper in the lacunae, outside the cortical region of nephrocytes in all three genotypes, with highest reduction in Baz-shRNA-expressing nephrocytes (Supplementary Fig. 3, arrows indicate slit diaphragms, arrow heads label basement membrane). Moreover, the morphology, in particular upon Baz- and PAR-6 knockdown, is strongly impaired with enlarged (almost vesicle-shaped) lacunae (Supplementary Fig. 3) and reduction of electron-dense vesicles (presumably late endosomes and lysosomes). Consequently, endocytosis efficiency of these cells is strongly reduced as estimated by the accumulation of secreted ANP-2xGFP molecules (Fig. 4F). Notably, similar to downregulation of basolateral polarity regulators, ex vivo endocytosis assays with FITC-Albumin showed a weaker phenotype (Supplementary Fig. 5J), with only nephrocytes expressing shRNA against aPKC displaying a significant reduction in short-term filtration and endocytosis. This is in line with observed disturbances in Rab5 and Rab7 (Supplementary Fig. 5G–I): in aPKC-shRNA-expressing nephrocytes, accumulation of ANP-2xGFP is strongly reduced and Rab5 and Rab7 are both found to be reduced to aggregates. Nephrocytes with knockdown of Baz display an almost normal Rab5 pattern, whereas the Rab7 staining suggests that late endosomes and lysosomes are smaller compared to control nephrocytes. Downregulation of PAR-6 results in normal Rab7 staining, whereas Rab5 is distributed all over the cell and not concentrated at the cortex and subcortical region like in control nephrocytes.

Apart from its effects on Sns localization, slit diaphragm assembly and endocytosis, we frequently observed a fusion of nephrocytes upon Baz, aPKC and PAR-6 knockdown (Fig. 4A–C), resulting in giant syncytia with multiple nuclei and fragmented Sns expression. This phenotype resembles that of Kirre-knockdown (Fig. 4D), indicating that loss of PAR/aPKC complex leads to defects in nephrocyte development and function.

### Cortical targeting of aPKC is sufficient to rescue Baz-dependent defects

The interaction between PAR-3/Baz and aPKC is a key mechanism in epithelial polarization and Baz is essential to target aPKC and its regulator PAR-6 to the apical junctions in classical epithelia and to the apical cortex of neuroblasts [54–56]. In mammalian cells, PAR-3 has been found to associate with the Nephrin/Neph1 complex [25, 26]; however, the in vivo impact of this interaction is not yet clear. To test whether the localization of Baz depends on the Neph1-homologue Kirre, we stained for endogenous Baz in nephrocytes expressing dsRNA against Kirre (Fig. 4D). As expected, downregulation of Kirre results in a disruption of Sns-labeled slit diaphragms as well as in a mislocalization of Baz from the cortex (Fig. 4D’), indicating that the Nephrin/Neph1 complex is essential for targeting of Baz to the cortex.

In nephrocytes with impaired Baz expression, aPKC and PAR-6 are not correctly localized to the cortex (Fig. 4A), suggesting a similar polarity hierarchy for nephrocytes as in epithelial cells. We investigated whether the defects observed in Baz-knockdown are due to impaired aPKC activity by co-expressing a constitutively active version of aPKC (aPKC∆N) and two different membrane-binding variants in Baz-shRNA-expressing nephrocytes: Neither the constitutively active (but due to loss of Baz cytosolic localized) aPKC∆N, nor an aPKC variant with a CAAX-motif, which facilitates transient membrane association due to farnesylation of the cysteine [36], were able to sufficiently rescue the developmental defects or disturbed Sns localization observed in Baz-shRNA-expressing nephrocytes (data not shown). In contrast, fusion of the pleckstrin-homology (PH) domain of phospholipase Cδ (PLCδ) to aPKC results in restored cortical localization of aPKC (Fig. 4G). PH (PLCδ) directly binds to phosphatidylinositol(4,5)-bisphosphate (PI(4,5)P2), thereby targeting the protein to the plasma membrane. Furthermore, co-expression of this transgene rescued the developmental phenotype as well as Sns localization in Baz-shRNA-expressing nephrocytes to a large extent (Fig. 4G compared to 4A, quantified in Fig. 4E).

These data demonstrate that membrane localization of aPKC is essential for development and Sns localization in nephrocytes and that the main function of Baz in this context is to target aPKC to the plasma membrane.

### Phosphorylation of Baz is essential for slit diaphragm maintenance

**Fig. 5:**
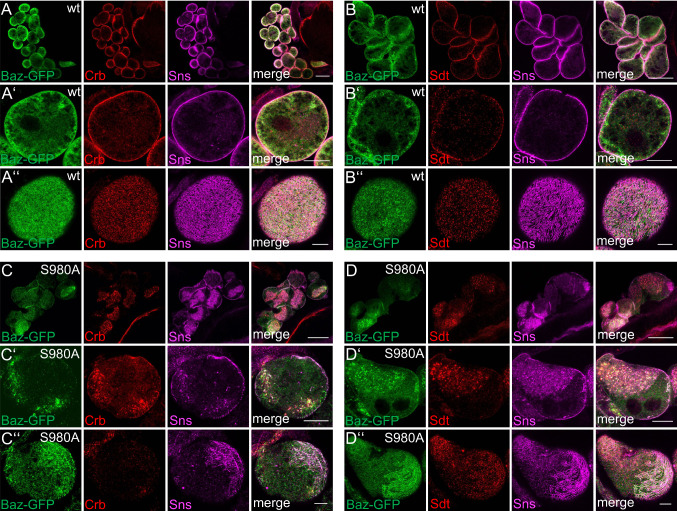
Phosphorylation of Baz is critical for its function in nephrocyte development and Sns localization. (**A**–**D**) Overexpression of wild-type GFP-Baz and GFP-Baz S980A in garland nephrocytes disrupts Crb-, Sdt- and Sns cortical localization. Scale bars are 25 µm in **A**–**D**, 10 µm in **A**′–**D**′ and 5 µm in **A**″–**D**″

Besides its targeting, Baz is also be phosphorylated by aPKC at the conserved Serine 980 [57] and overexpression of a non-phosphorylatable variant of Baz results in severe polarity defects in epithelia but not in neuroblasts due to impaired dissociation of Baz and Sdt [58]. Introduction of GFP-Baz S980A in nephrocytes results in strong mislocalization of the mutant protein as well as of Sdt and Crb (Fig. 5). We have recently shown that Sdt as well as Crb are essential for slit diaphragm assembly/maintenance [5, 6]. Consequently, disruption of cortical Sdt/Crb by overexpression of Baz S980A leads to a reduction of cortical Sns localization (Fig. 5C’ and D’ compared to A’ and B’) and strongly disturbed fingerprint pattern of Sns-marked slit diaphragm strands at the surface of nephrocytes (Fig. 5C’’ and D’’ compared to A’’ and B’’). Instead, Sns frequently accumulates in intracellular aggregates together with GFP-Baz S980A, Sdt and Crb (C’ and D’). In contrast, overexpressed wild-type GFP-Baz localizes similar to the endogenous protein at the cortex of nephrocytes, which show no obvious defects in Sns, Sdt or Crb localization (Fig. 5A’, B’). Notably, overexpression of Baz S980A but not of wild-type Baz results in frequent fusion of nephrocytes (Fig. 5C, D compared to 5A, B), similar to nephrocytes with impaired Baz expression (Fig. 4A).

## Discussion

**Fig. 6:**
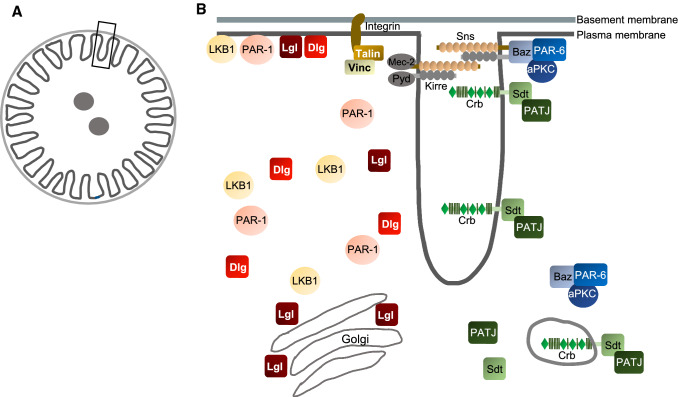
Schematic illustration of localization of polarity regulators in garland nephrocytes

Although nephrocytes are an emerging model to study podocyte development and podocyte-associated diseases, little is known about its structural characteristics regarding polarity protein expression and localization. In this study, we systematically analyzed the subcellular localization of classical polarity regulators (summarized in Fig. 6) with super resolution expansion microscopy as well as the functional consequences of their knockdown. We found that—like in podocytes—the slit diaphragm of nephrocytes and its “junctional zone” seem to separate the basal from the apical plasma membrane domain at the expense of the lateral domain. Proteins of the PAR/aPKC and Crb-complex are localized to the cortex with some but not exclusive colocalization with Sns but do not show the typical fingerprint pattern typical for slit diaphragms as seen in Sns stainings. This is in line with previous results obtained from immune electron microscopy of Crb-GFP, which revealed a localization of Crb at slit diaphragms as well as along lacunae and on vesicles [5]. Similar to podocytes, integrin complex components (Integrin β1, Talin, Vinculin) localize adjacent (and substantially overlapping) to Sns-marked slit diaphragms, enabling the anchorage of the nephrocyte to the surrounding basal membrane. Surprisingly, polarity regulators, which are localized to the lateral membrane in classical epithelia (Dlg, Lgl, PAR-1, LKB1) show a rather weak association with the cortex of nephrocytes and only some overlap with Sns-marked slit diaphragms. Instead, they accumulate in intracellular vesicles, which we identified as trans-golgi tubules for Lgl. Some GFP-LKB1-positive vesicles were positive for Rab5 and we further found some overlap of PAR-1, LKB1 but not of Dlg with the endocytosis regulator Megalin, suggesting these structures to be vesicles budding from lacunae. However, the character of other PAR-1/LKB1/Dlg-positive vesicular structures, which do not co-stain with Megalin still remains elusive. One hypothesis would be that it represents vesicles budding from lacunae, which do not assemble Megalin. Indeed, Megalin was reported to be not essential for endocytosis in nephrocytes [15, 59]. Notably, we observed a substantial colocalization with the early endocytosis marker Rab5 only in case of some GFP-LKB1-positive structures (and even fewer for PAR-1 and Dlg). Moreover, endocytosed ANP-2xGFP is stored in vesicular structures, of which many but not all are coated with Rab5 (early endosomes) or Rab7 (late endosomes/lysosomes). An explanation of these discrepancies would be a Rab5-independent endocytosis mechanism for vesicle budding from lacunae, which was reported in yeast [60].

Functionally, basolateral polarity regulators are essential for slit diaphragm formation/maintenance and their downregulation disrupts regular Sns-strands, which was most prominent in case of Scrb and LKB1. Notably, *SCRB* knockout mice do not display any defects in podocyte morphology or function although *SCRB* is an essential gene in mice [61]. It is still unclear, whether this is due to redundancy with another protein or whether basolateral determinants are not essential in podocyte development. In nephrocytes, we found strong defects in Sns localization in all, Scrb-, Lgl- and Dlg-knockdown cells. As we did not observe a frequent colocalization of these proteins with Sns, which might explain these defects by physical interaction(s), we speculate that these proteins regulate Sns localization at the cortex of nephrocytes indirectly by regulating the endocytosis- and recycling machinery, which was shown for Lgl in *Drosophila* imaginal discs [62, 63]. Notably, in this system, Lgl associates with early- and late endosomes as well as lysosomes, which we did not see in nephrocytes. However, an inhibiting role of Lgl in processing endocytic vesicles could explain the lack of endocytosis defects in nephrocytes: reduction of slit diaphragms in Lgl-knockdown cells results in decreased endocytosis, whereas concomitant reduction of its inhibitory effect on processing vesicles compensates the decreased endocytosis.

For the PAR/aPKC complex, our results confirm a crucial role of these proteins in regulating slit diaphragm assembly/maintenance as well as endocytosis, which was already shown in knockout mice studies [25–30]. Notably, our rescue data show that the main function of Baz in nephrocytes is the cortical recruitment of aPKC, as membrane-bound aPKC can compensate for knockdown of Baz. Impaired phosphorylation of Baz by aPKC results in strong polarity defects in epithelial cells by outcompeting Crb for Sdt binding [58]. Sdt (as well as Crb) are essential for slit diaphragm assembly/maintenance and ultrastructure of nephrocytes [5, 6], explaining the dominant negative phenotypes upon overexpression of a non-phosphorylatable version of Baz.

Taken together, our results suggest that nephrocytes, although they are not derived from epithelial progenitor cells, exhibit a distinct apical-basal polarity regulated by classical polarity regulators, which is essential for slit diaphragm assembly/maintenance and endocytosis and thus for the function of nephrocytes.

### Supplementary Information

Below is the link to the electronic supplementary material.

Supplementary file1 Supplementary figure 1. Co-stainings of basolateral cell polarity determinants with endosomal markers. (A-D) Endogenous Lgl, GFP-traps of PAR-1 and Dlg as well as GFP-LKB1 expressed from its endogenous promoter were co-stained with marker for early endosomes (Rab5, Hrs), late endosomes/lysosomes (Rab7) and recycling endosomes (Rab11). Scale bars are 5µm (PDF 670 KB)

Supplementary file2 Supplementary figure 2. Co-stainings of basolateral cell polarity determinants with Golgi markers. (A-D) Endogenous Lgl, GFP-traps of PAR-1 and Dlg and GFP-LKB1 expressed from its endogenous promoter were co-stained with marker for cis-Golgi (GS28, GM130), trans Golgi network (Vti1b, p230) and Megalin (Mgl). Scale bars are 5µm (PDF 959 KB)

Supplementary file3 Supplementary figure 3. Ultrastructure of nephrocytes with downregulated PAR-complex components. (A-D) Transmission electron microscopy of garland nephrocytes of third instar larvae expressing Control shRNA (A) and shRNA/dsRNA against Baz (B), aPKC (C) and PAR-6 (D). (E) Quantification of slit diaphragms (SD)/µm for Baz, aPKC and PAR-6 shRNA/dsRNA-expressing cells. Slit diaphragms are marked with arrows, basement membrane is marked with arrow heads. Scale bars are 1µm (PDF 288 KB)

Supplementary file4 Supplementary figure 4. Clusters of nephrocytes with knockdowns of basolateral cell polarity regulators show a different morphology. (A-E) Overviews of clusters of garland nephrocytes expressing Control shRNA and shRNA/dsRNA against Dlg (A), Scrb (B), Lgl (C), PAR-1 (D) and LKB1 (E) partly exhibit a fused morphology in addition to the mislocalization of Sns and Vinculin/Talin. Scale bars are 25µm in A-E (PDF 1026 KB)

Supplementary file5 Supplementary figure 5. Accumulation of ANP-2xGFP in garland nephrocytes with impaired expression of polarity regulators. (A-I) Garland nephrocytes of ANP-2xGFP secreting larvae expressing the indicated shRNA/dsRNA were stained against Rab5 and Rab7. (J) Quantification of ex-vivo endocytosis of garland nephrocytes incubated with FITC-Albumin (n > 50). Error bars are standard error of the means. Significance was determined by Kruskal-Wallis test and Dunn’s correction: **** p < 0.0001, *** p<0.001, ** p<0.01,* p<0.05. n.s. not significant. Scale bars are 5µm (PDF 619 KB)

## Data Availability

All data generated or analyzed during this study are included in this published article and its supplementary information files. Reagents used in this publication (e.g., fly stocks and antibodies) will be provided upon request.
