# Supplementary figures and images for: Apical-basal polarity regulators are essential for slit diaphragm assembly and endocytosis in Drosophila nephrocytes
Source: Cell Mol Life Sci. 2021 Mar 2;78(7):3657–72. doi: 10.1007/s00018-021-03769-y (PMC8038974; doi:10.1007/s00018-021-03769-y)

Figure S2

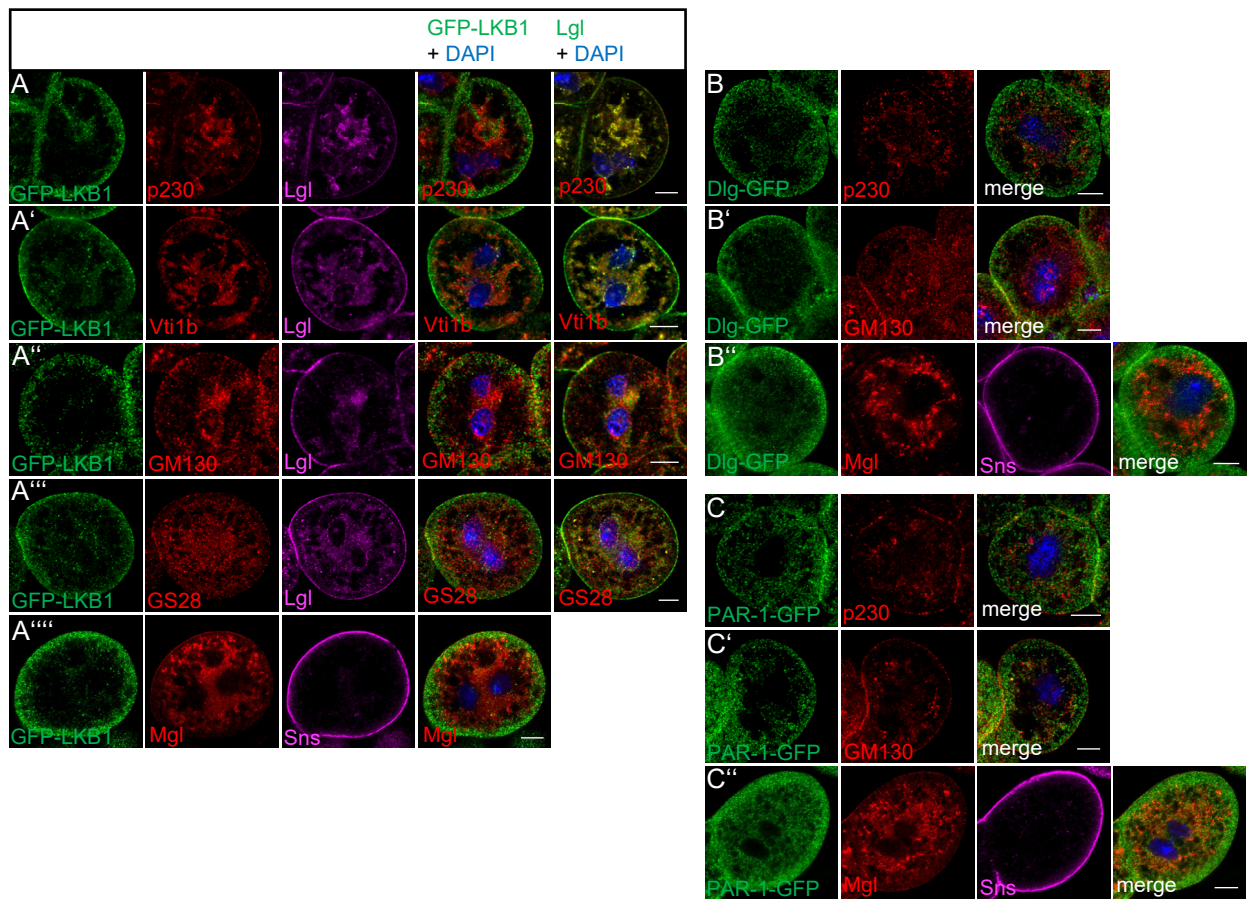

Supplement: Supplementary file 1 — Supplementary file1 Supplementary figure 1. Co-stainings of basolateral cell polarity determinants with endosomal markers. (A-D) Endogenous Lgl, GFP-traps of PAR-1 and Dlg as well as GFP-LKB1 expressed from its endogenous promoter were co-stained with marker for early endosomes (Rab5, Hrs), late endosomes/lysosomes (Rab7) and recycling endosomes (Rab11). Scale bars are 5µm (PDF 670 KB) [file 18_2021_3769_MOESM2_ESM.pdf]

Figure 6

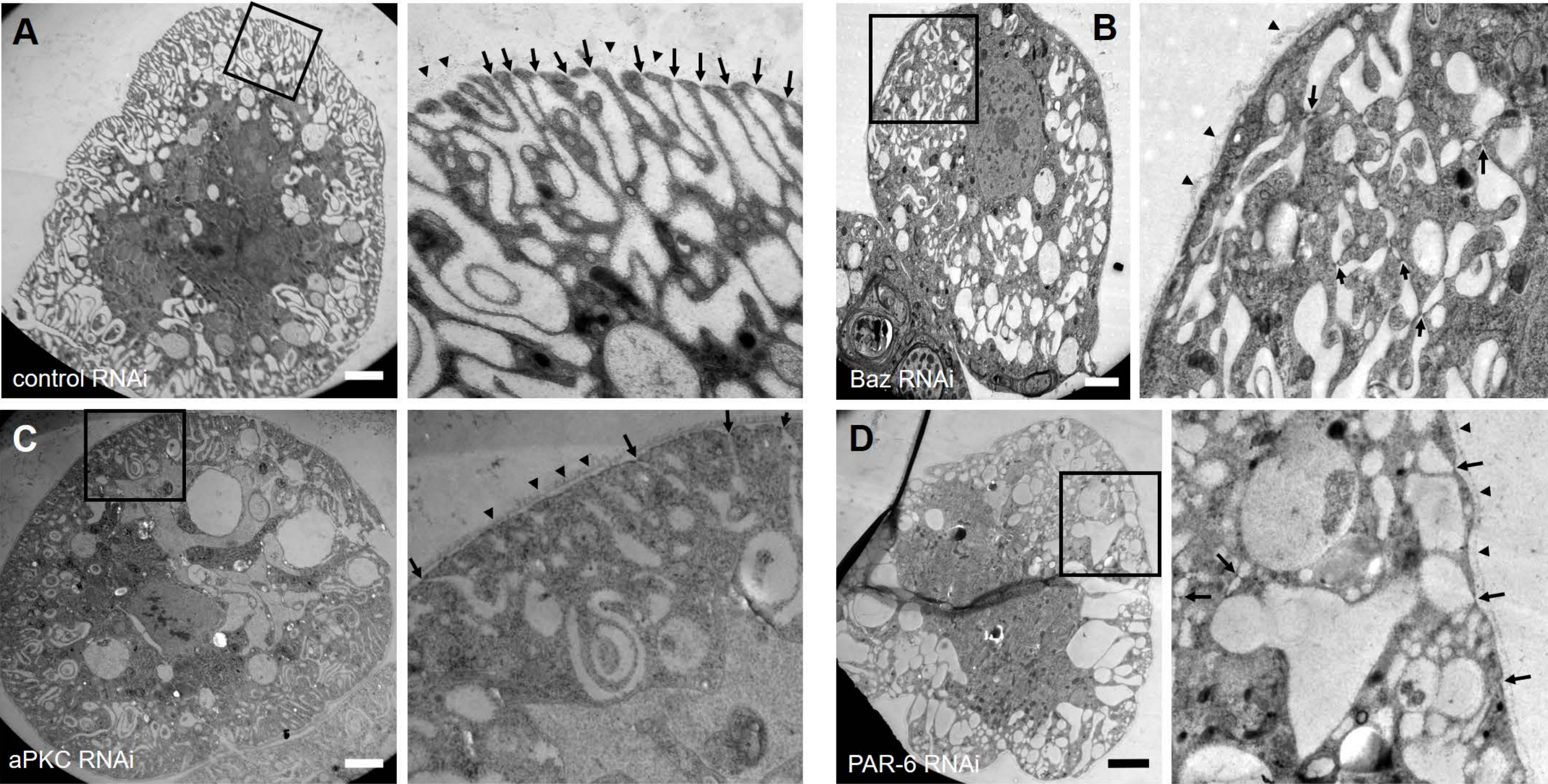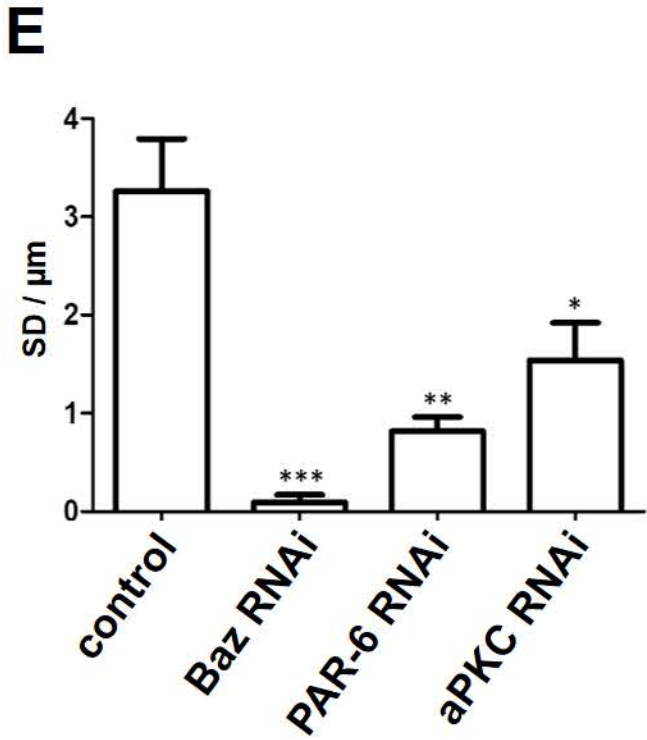

Supplement: Supplementary file 2 — Supplementary file2 Supplementary figure 2. Co-stainings of basolateral cell polarity determinants with Golgi markers. (A-D) Endogenous Lgl, GFP-traps of PAR-1 and Dlg and GFP-LKB1 expressed from its endogenous promoter were co-stained with marker for cis-Golgi (GS28, GM130), trans Golgi network (Vti1b, p230) and Megalin (Mgl). Scale bars are 5µm (PDF 959 KB) [file 18_2021_3769_MOESM3_ESM.pdf]

Figure S5

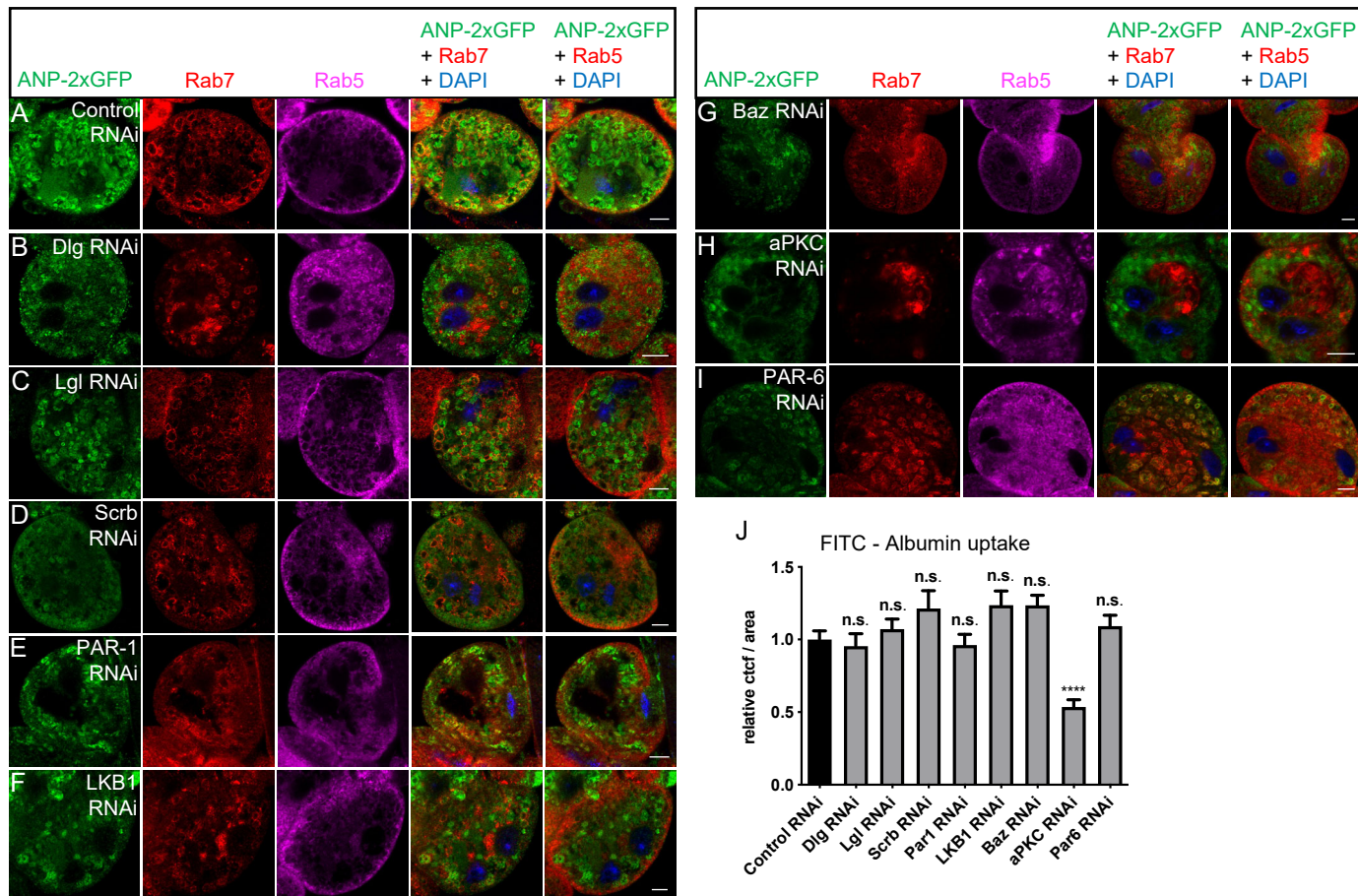

Supplement: Supplementary file 3 — Supplementary file3 Supplementary figure 3. Ultrastructure of nephrocytes with downregulated PAR-complex components. (A-D) Transmission electron microscopy of garland nephrocytes of third instar larvae expressing Control shRNA (A) and shRNA/dsRNA against Baz (B), aPKC (C) and PAR-6 (D). (E) Quantification of slit diaphragms (SD)/µm for Baz, aPKC and PAR-6 shRNA/dsRNA-expressing cells. Slit diaphragms are marked with arrows, basement membrane is marked with arrow heads. Scale bars are 1µm (PDF 288 KB) [file 18_2021_3769_MOESM5_ESM.pdf]

Figure S1

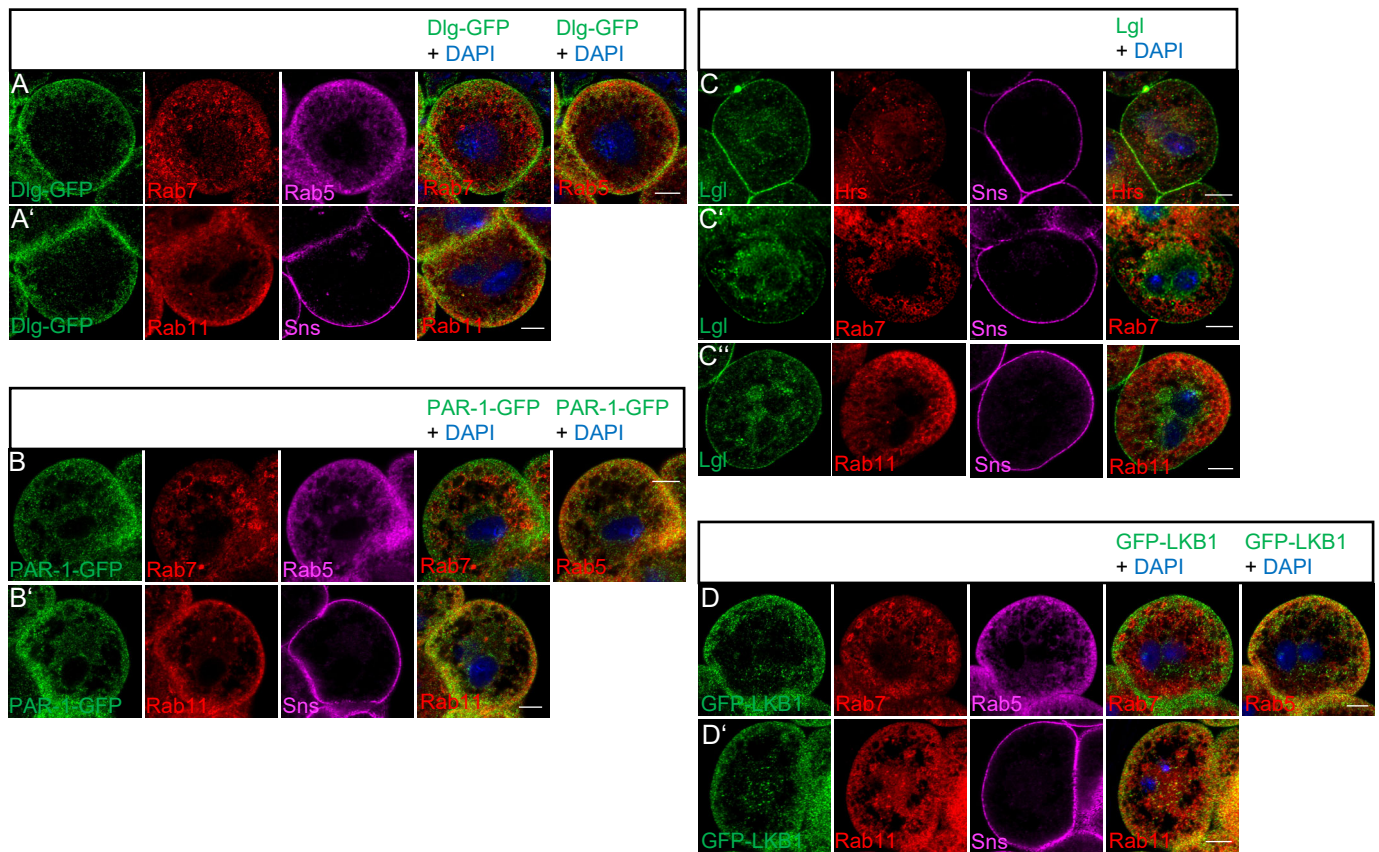

Supplement: Supplementary file 4 — Supplementary file4 Supplementary figure 4. Clusters of nephrocytes with knockdowns of basolateral cell polarity regulators show a different morphology. (A-E) Overviews of clusters of garland nephrocytes expressing Control shRNA and shRNA/dsRNA against Dlg (A), Scrb (B), Lgl (C), PAR-1 (D) and LKB1 (E) partly exhibit a fused morphology in addition to the mislocalization of Sns and Vinculin/Talin. Scale bars are 25µm in A-E (PDF 1026 KB) [file 18_2021_3769_MOESM1_ESM.pdf]

Figure S4

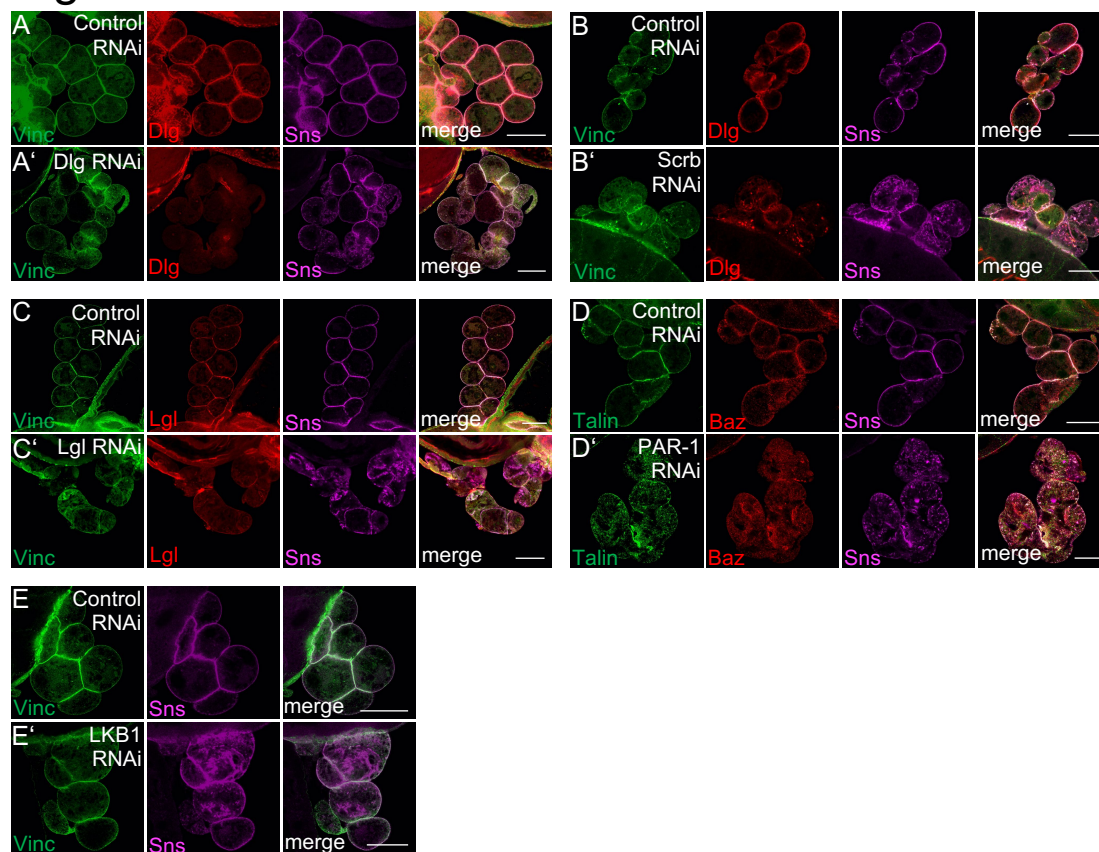

Supplement: Supplementary file 5 — Supplementary file5 Supplementary figure 5. Accumulation of ANP-2xGFP in garland nephrocytes with impaired expression of polarity regulators. (A-I) Garland nephrocytes of ANP-2xGFP secreting larvae expressing the indicated shRNA/dsRNA were stained against Rab5 and Rab7. (J) Quantification of ex-vivo endocytosis of garland nephrocytes incubated with FITC-Albumin (n > 50). Error bars are standard error of the means. Significance was determined by Kruskal-Wallis test and Dunn’s correction: **** p < 0.0001, *** p<0.001, ** p<0.01,* p<0.05. n.s. not significant. Scale bars are 5µm (PDF 619 KB) [file 18_2021_3769_MOESM4_ESM.pdf]
